# The regulatory role of miR-107 in Coxsackie B3 virus replication

**DOI:** 10.18632/aging.103488

**Published:** 2020-07-16

**Authors:** Min Yao, Chi Xu, Hongxing Shen, Tingjun Liu, Xiuping Wang, Chen Shao, Shihe Shao

**Affiliations:** 1 School of Medical Science and Laboratory Medicine, Jiangsu University, Zhenjiang 212013, Jiangsu, China; 2 Marshall International Center for Digestive Diseases, Shanghai East Hospital, Tongji University, Shanghai 200120, China; 3 Department of Cardiology, Affiliated Hospital of Jiangsu University, Zhenjiang 212013, Jiangsu, China

**Keywords:** CVB3, lncRNA-004787, MiR-107, NFκB, p-NFκB

## Abstract

Coxsackie B3 virus (CVB3) is a member of small RNA viruses that belongs to the genus Enterovirus of the family Picornaviridae and CVB3 is the main pathogen of acute and chronic viral myocarditis. In this study RT-qPCR was used to determine the expression of miR-107 in CVB3-infected and uninfected HeLa cells. The experimental results show that the level of miR-107 began to rise at 4 h after the infection, and significantly boosted at 6 h. Based on the results of this experiment, we consider that miR-107 expression is related to CVB3 infection. In order to further clarify the effect of miR-107 in the process of CVB3 infection, we studied the effect of miR-107 upstream and downstream target genes on CVB3 replication. Levels of the target RNAs were detected by RT-qPCR after CVB3 infection, and the expression of CVB3 capsid protein VP1 by western blot analysis. Then the virus in the supernatant was quantitated via a viral plaque assay, reflecting the release of the virus. The experimental results showed that miRNA-107 expression is associated with CVB3 replication and proliferation, while KLF4 and BACE1 as the downstream of miR-107 weakened CVB3 replication. Overexpressions of KLF4 and BACE1 negatively regulated CVB3 replication, this effect on CVB3 was completely opposite to that of miR-107. Further experiments revealed that the upstream lncRNA004787, a new lncRNA that had not been reported, was located on chromosome 5, strand - from 37073250 to 37070908 (genome assembly: hg19). We sequenced and studied lncRNA004787 and found that it partially inhibited CVB3 replication. This prompted us to speculate that lncRNA004787 probably impacted the replication by other means. In conclusion, miR-107 interfered with CVB3 replication and release.

## INTRODUCTION

Coxsackie virus is a positive-sense single-stranded RNA virus belonging to the genus Enterovirus of the family Picornaviridae [1]. When invading the human body, it causes upper respiratory tract infections, resulting in fever, cough and other cold symptoms. Severe infection even leads to diseases such as myocarditis, myositis, encephalitis and meningitis. Among the subgroups of coxsackie virus, CVB3 is the main pathogen of human viral myocarditis [2].

MicroRNA (miRNA), a non-protein-coding RNA, is widely distributed in the cytoplasm, predominating the largest family of mammalian regulatory genes. It should be the function of nearly half of human genes via interfering with endogenous RNA. Recently, it has been reported that miRNAs may disturb the interaction in various ways after CVB3 invades the host [3–6], which has become a research hotspot.

One of the main mechanisms currently known is the competitive binding of lncRNA with miRNA, called competing endogenous (ceRNA) [7, 8]. LncRNA is categorized as a member of the non-coding RNA (ncRNA) family which does not encode proteins. NcRNAs encompass a variety of subgroups, such as miRNAs, lncRNAs and circular RNAs (circRNAs), of which lncRNAs refer to a generic term for ncRNA molecules of over 200 nt in length [9, 10]. As a significant gene regulator, lncRNA regulates expressions of target genes at different levels in their epigenetics, transcription and post-transcription. Over the past few years, more reports have surged over the mutual regulation between lncRNAs and miRNAs, and the underlying mechanisms behind the interaction have been explored in a majority of cancers [11–13]. Evidence shows that long non-coding RNA (lncRNA) has a certain degree of influence on the replication and release of viruses [14, 15]. Recent studies have proved that viruses or interferons (IFNs) can stimulate expressions of a variety of lncRNAs, viewed as regulators of antiviral innate immunity [16]. Despite many researches on correlations between miRNAs and viral replication, which have been well established and frequently reported, those between lncRNA and viral replication are still at the embryonic stage. At present, relevant studies have shown that lncRNA indirectly regulates viral replication at the cellular level [17, 18, 19], but whether the mechanism by which lncRNA can directly interfere with the genome sequence of the virus is unclear. CVB3 is a common pathogen of viral myocarditis. lncRNA regulates the viral replication, proliferation and release in host cells. The effect of lncRNA-miRNA in the replication and pathogenesis of CVB3 remain unclear. This experiment uses miR-107 as an entry point, focusing on the effects and mechanisms of miR-107-related target molecules on the replication and diffusion of CVB3, and initially exploring the effect of miR-107 upstream gene lncRNA004787 on CVB3 replication. However, as the pair is expected to become a new biomarker for prognosis, even diagnosis in clinical practice, in-depth explorations on the mechanism between lncRNA and miRNA are required.

## RESULTS

### MiR-107 was associated with CVB3 replication

**Figure 1:**
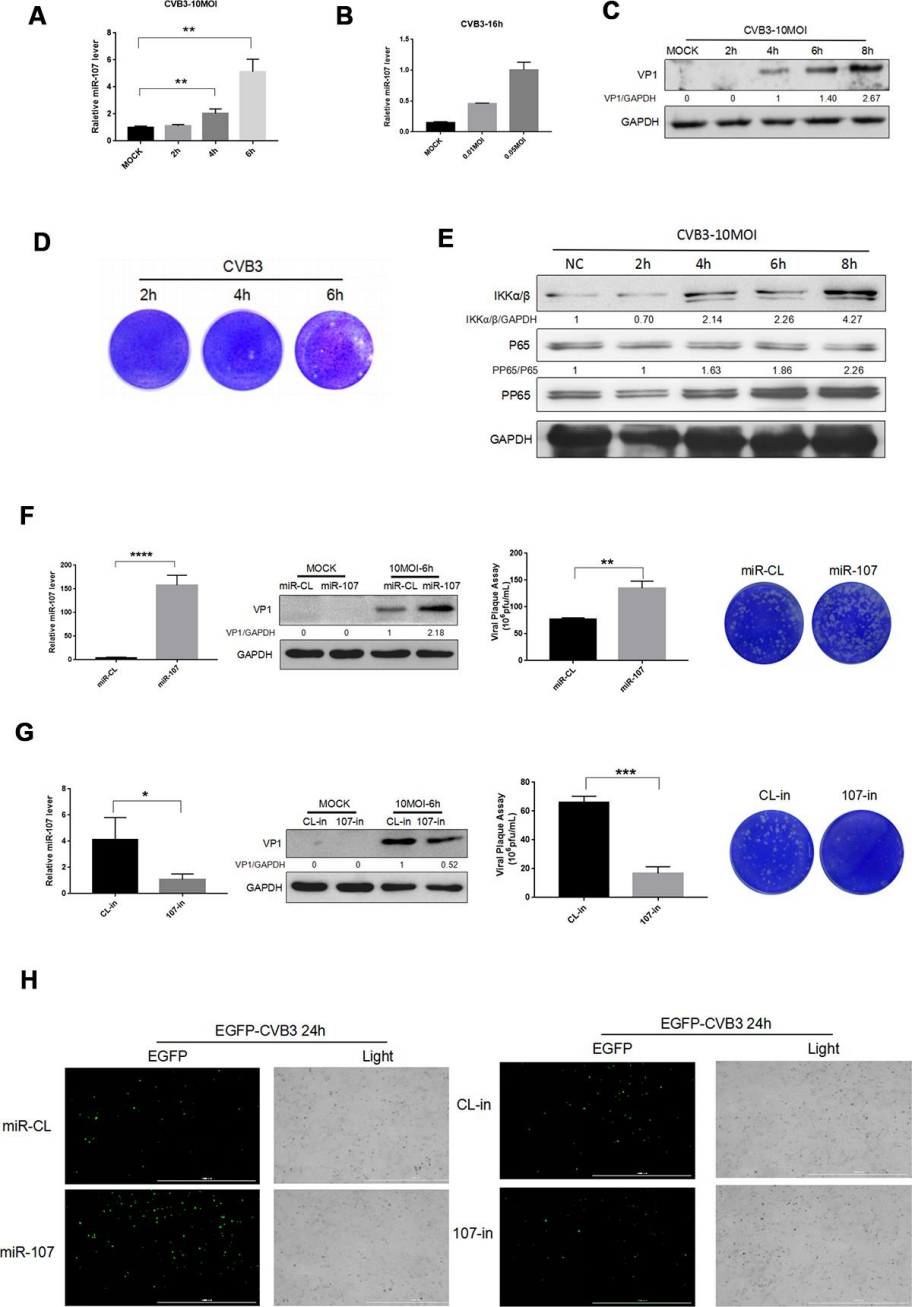
(**A**–**B**) Hela cells were infected with CVB3 with specified titers at planned time points. Total RNA of the cells were extracted and miR-107 was detected using real-time PCR. (**C**–**E**) Hela cells were infected at a certain concentration of CVB3, and viral replication at different time points was observed. (**F**) The transfection efficiency of miR-107 mimics was analyzed using RT-qPCR, and western blot reflected viral replication at a protein level. Viral titers were determined by plaque assay. (**G**) The transfection efficiency of miR-107 inhibitors was detected by RT-qPCR, and western blot reflected virus replicated at a protein level. Viral titers were determined by plaque assay. (**H**) The replication level of EGFP-Iabeled CVB3 was observed in Hela cells which had been transfected with mimics107 or 107-in (magnification, 4×).

The miRNAs associated with CVB3 replication were retrieved on the relevant websites using *ViTa-miRNA-targeted virus*, and selected the higher-rated factor miR-107 for our experiments. To investigate the interaction between miR-107 and CVB3 replication and proliferation in the process of the viral infection, we infected Hela cells with CVB3 and determined the level of miR-107 in different phases of the infection. Regarding cellular inflammatory responses that resulted from CVB3 infection, we selected the most representative NF*κ*B signaling pathway in our study. In the early stage of CVB3 infection (0-2 h), nonsignificant difference was found in the expression of miR-107 compared with the blank control group. The level of miR-107 began to rise at 4 h after the infection, and significantly boosted at 6 h (Figure 1A). The level of miR-107 was elevated in Hela cells in a dose-dependent manner after 16 h of infection with low-concentration virus (Figure 1B). The VP1 antibody was used to reflect CVB3 replication. It was found that the replication which was initiated in Hela cells at 4 h after infection revealed a drastic increase at 6 h (Figure 1C). Viral plaque experiments showed that the number of viral plaques increased significantly in Hela cells after 6 h of infection with CVB3 (Figure 1D). Besides, expressions of NF*κ*B and p-NF*κ*B were analyzed by western blot. As a result, the upward trends of NF*κ*B and p-NF*κ*B to different extents were observed. The p-NF*κ*B / NF*κ*B ratio increased by 86% in Hela cells infected with CVB3 for 6 h compared with those after 2 h of infection featuring increasing levels as the process of virus infection moved forward (Figure 1E).

To investigate the effect of miR-107 expression on host cells and viral replication, we preliminarily transfected Hela cells with mimics-107 and mimics-nc, and then observed differences in CVB3 replication and proliferation. RT-qPCR data revealed that mimics-107 facilitated the expression of miR-107 in the cells compared with the control group at 48 h after cell transfection (Figure 1F). We observed that the expression level of CVB3 VP1 was positively correlated with the concentration of mimics-107 after 6 h of infection (Figure 1F). Virus plaque assay even confirmed that miR-107 significantly enhanced the release of virus progeny, and this effect was more evident with the increase of miR-107 concentration.

We applied miRNA inhibitors for suppressing the level of endogenous miR-107, and RT-qPCR presented that the miR-107 inhibitor (107-in) successfully repressed the expression of miR-107 in the transfected Hela cells compared with the control group (Figure 1G). The western blot analysis revealed that 107-in also significantly suppressed the expression of VP1 (Figure 1G). The virus plaque assay showed that 107-in significantly thwarted the release of the virus. All these suggested that miR-107 evidently promoted CVB3 replication (Figure 1G). In addition, EGFP-CVB3 was used to infect Hela cells that had been transfected with mimics-107 or 107-in. The fluorescence intensity of the mimics-107 group (green) was significantly enhanced, while that of the 107-in group was slightly reduced (Figure 5H).

### Target gene KLF4 was associated with CVB3 replication and proliferation

**Figure 2:**
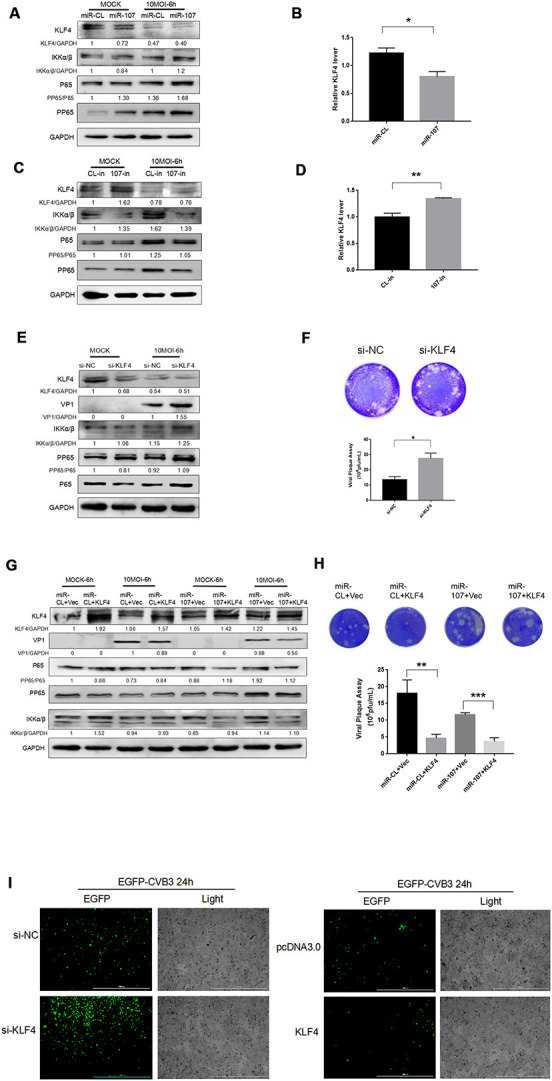
(**A**–**D**) MiR-107 targeted KLF4, enhanced phosphorylation of NFκB and promoted replication of CVB3. Hela cells was infected with CVB3 after transfection with miRNA mimics or inhibitors. Cellular proteins were detected by western blot. RT-qPCR was performed to determine the mRNA level of KLF4. (**E**) KLF4 knockdown facilitated the phosphorylation of NFκB. Hela cells which were initially transfected with KLF4 siRNA were infected with CVB3. Protein expressions of NFκB and p-NFκB were detected by western blot analysis. (**F**, **H**) Viral titers were determined by plaque assay. (**G**) The overexpression of KLF4 partially suppressed the promoting effect of miR-107 on VP-1 synthesis and the phosphorylation of NFκB. Hela cells that were co-transfected with miRNA mimics and KLF4 plasmids or empty vector (Vec) were infected with CVB3 afterward. The activity of NFκB signaling was analyzed by western blot. (**I**) The replication level of EGFP-Iabeled CVB3 was observed in Hela cells which had been transfected with si-KLF4 or pcDNA-KLF4 (magnification, 4×).

KLF4 is a target gene of miR-107, receiving increasing attentions on its role in promoting cancer cell metastasis. However, as a downstream target gene of miR-107, KLF4 effect on CVB3 replication has not been studied. Clarifying their interactions may reveal more interesting mechanisms behind them. To this end, we adopted miR-107 mimics (10 nM) and 107-in (10 nM) to explore their effects on expressions of KLF4 and NF*κ*B and CVB3 replication. It was found that in the experimental group transfected with miR-107, the level of KLF4 significantly declined in both CVB3-infected and uninfected Hela cells (Figure 2A). Compared with miR-CL, the level of KLF4 approximately dropped by 50% in the cells transfected with miR-107 mimics. However, the level of KLF4 significantly rose in 107-in experimental group compared with CL-in group (Figure 2C). KLF4 mRNA expression was also observed (Figure 2B, 2D), and the KLF4 level decreased during CVB3 infection. MiR-107 mimics provoked the inflammatory responses in Hela cells with mounting levels of NF*κ*B and p-NF*κ*B (Figure 2A). MiR-107 inhibitors moderately quenched the inflammatory responses caused by CVB3 infection, accompanied by down-regulations of NF*κ*B and p-NF*κ*B accordingly (Figure 2C).

Si-klf4 was adopted to knock down KLF4 gene. The western blot analysis showed that KLF4 knockdown promoted the replication of CVB3 (Figure 2E). We found that KLF4 siRNA resulted in approximately 70% inhibition of KLF4 RNA expression and facilitated the phosphorylation of NF*κ*B and the synthesis of VP-1 during CVB3 infection (Figure 2E). The virus plaque assay revealed that si-KLF4 promoted the release of the virus (Figure 2F). To further clarify the interaction and integrated effect between KLF4 and miR-107 on CVB3 replication, Hela cells were co-transfected with KLF4 and miR-107 for 48 hours. Western blot was used to determine the expression of VP1. The results showed that the KLF4 level of KLF4 + miR-107 in the experimental group was significantly lower than that of the control group (KLF4 + miR-CL), while the expression of VP1 was significantly higher than that of the control group (Figure 5G). In addition, EGFP-CVB3 was used to infect Hela cells that had been transfected with si-KLF4 or the overexpression plasmid PCDNA3.0-KLF4. After 24 hours of infection, compared with the control group, the fluorescence intensity of the KLF4 group (green) was significantly reduced and the fluorescence intensity of the si-KLF4 group was significantly enhanced under the fluorescence microscope (Figure 5I).

### Target gene BACE1 was associated with CVB3 replication and proliferation

**Figure 3:**
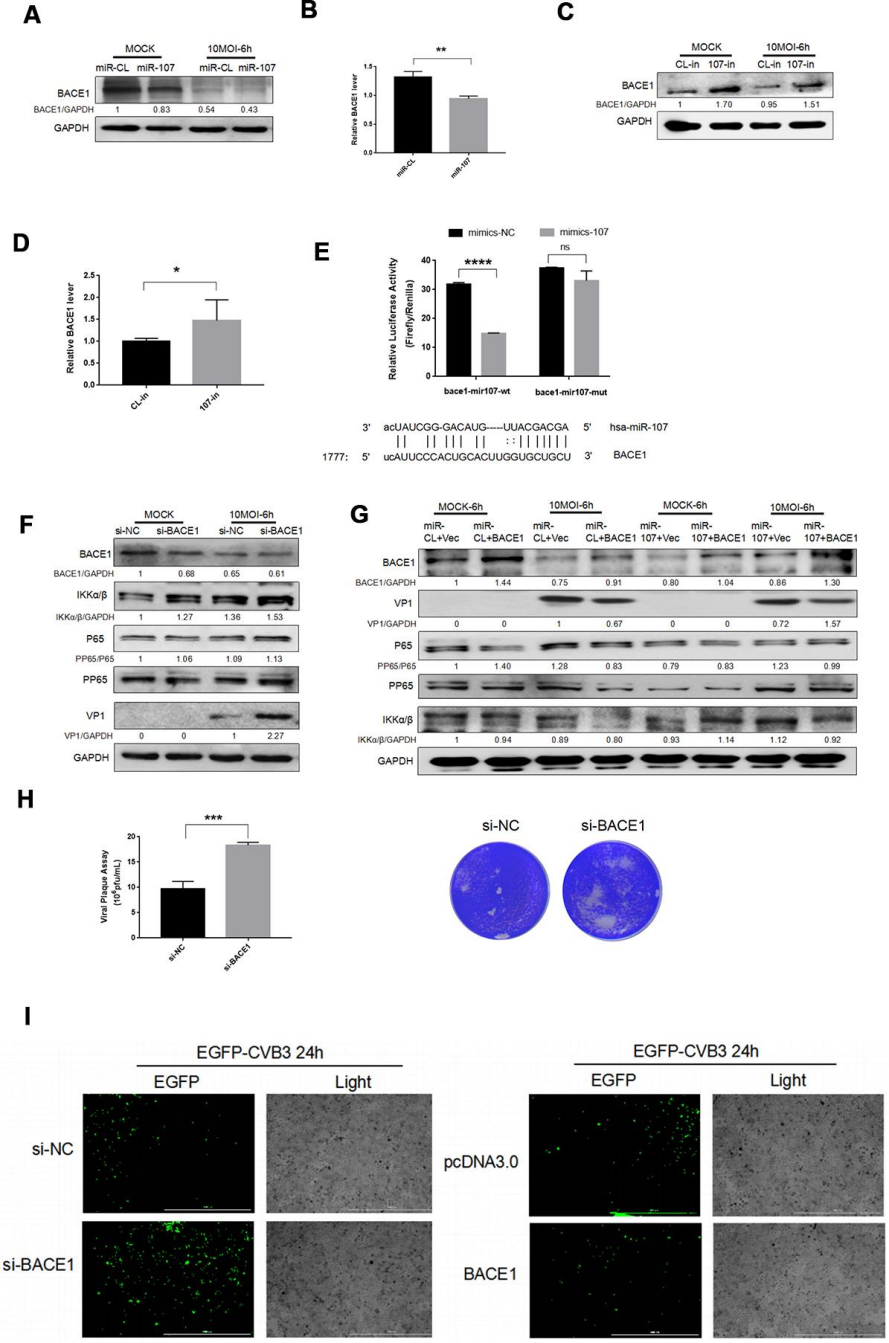
(**A**–**D**) MiR-107 targeted BACE1. CVB3 was infected with Hela cells after the transfection with miRNA mimics or inhibitors. Cellular proteins were used for western blot analysis. RT-qPCR was performed to determine the mRNA level of BACE1. (**E**). Luciferase was adopted to validate downstream BACE1 of miR-107, and Hela cells were co-transfected with BACE1-wt/mut and miR-107 or miR-CL. The activity of firefly and leucinase was calculated by the ratio of double luciferase to leucinase. (**F**) BACE1 knockdown promoted the phosphorylation of NFκB. Hela cells that were transfected with si-BACE1 were infected with CVB3 virus afterward. Cellular protein expressions of NFκB and p-NFκB were detected by western blot. (**G**) The overexpression of BACE1 partially reversed the facilitating effect of miR-107 on the production of VP-1 and the phosphorylation of NFκB. The activity of NFκB signaling was detected using western blot. (**H**) Viral titers were determined by plaque assay. (**I**) The replication level of EGFP-Iabeled CVB3 was observed in Hela cells which had been transfected with siBACE1 or pcDNA-BACE1 (magnification, 4×).

We retrieved other potential targets of miR-107 in miRtarbase, a miRNA target database, and BACE1 was screened out in a number of predicted target genes. To investigate the role of miR-107 in regulating BACE1, we transfected Hela cells with miR-107 mimics or miR-CL and detected BACE1 level in distinct experimental groups. The protein expression of BACE1 was significantly suppressed after the transfection with miR-107 mimics compared with the control group (Figure 3A). The BACE1 mRNA expression level was significantly elevated using the miR107 inhibitor, 107-in, compared with the control group (Figure 3C). After that, the changes of BACE1 mRNA expression were verified (Figure 3B, 3D), and it was found that miR-107 significantly inhibited the expression of BACE1. In addition, our study found that BACE1 expression was slightly suppressed after CVB3 infection compared with the negative control group (Figure 3A). To verify whether BACE1 was a true target of miR-107, we constructed luciferase reporter genes, including wild-type and mutant vectors of the 3’ UTR of the targeted gene BACE1. Seed matching mutations were introduced by changing four base pairs in a gene sequence. Luciferase experiments showed that miR-107 significantly suppressed luciferase activity of the BACE1 reporter gene (Figure 3E), indicating that BACE1 was a novel target gene of miR-107.

To investigate the role of BACE1 in the miR-107-mediated NF*κ*B signaling pathway, we detected NF*κ*B expression after CVB3 infection. We first confirmed that BACE1 knockdown promoted expressions of NF*κ*B and p-NF*κ*B (Figure 3F). However, the overexpression of BACE1 inhibited expressions of the two proteins (Figure 3G). This indicated that BACE1 as the target gene of miR-107 provoked inflammatory responses in CVB3-infected Hela cells. Consistent with the expression of KLF4, the virus plaque assay showed that BACE1 promoted CVB3 progeny release (Figure 3H). Similarly, we separately transfected miR-107 mimics and the overexpressed plasmid BACE1 in a bid to explore the interaction between miR-107 and BACE1. The corresponding results showed that the overexpression of BACE1 partially curbed the promoting effect of miR-107 mimics on viral replication (Figure 3G). The fluorescent virus experiment also illustrated this conclusion (Figure 3I).

These results suggested that miR-107 targeted BACE1 and further promoted virus-induced cell death and CVB3 progeny release by regulating NF*κ*B signaling pathway.

### Interaction analysis between lncRNA004787 and miR-107

**Figure 4:**
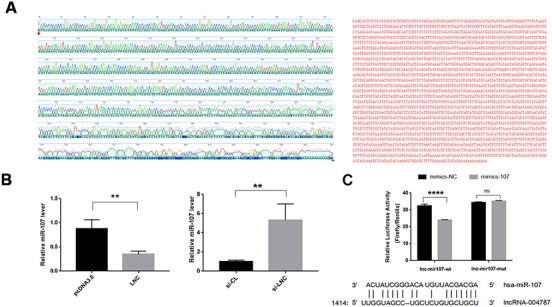
(**A**) The full length sequencing of IncRNA004787 and partial sequencing signals were presented. (**B**) RT-qPCR verified that IncRNA004787 targeting to miR-107 manifested a biological role of their interaction. (**C**) Luciferase was used to verify the targeting of IncRNA004787 to miR-107.

To further elucidate the regulatory mechanism of miR-107, we performed base pairing of the known partial sequences of lncRNA004787 and miR-107 at the relevant sites. The full length of lncRNA004787 was sequenced using TaKaRa Taq. Part of the sequencing map and the full length sequencing of lncRNA004787 were presented in Figure 4A. We further identified the target binding sites between lncRNA004787 and miR-107 using the dual fluorescein reporter gene approach. We predicted potential sites for direct binding of lncRNA004787 to miR-107 from TargetScan website, and the results showed a binding site near the 1414-1437 bp position. We mutated this site and performed a dual luciferase assay. When the 𲀲-UTR of the lncRNA004787 mRNA was involved in the luciferase transcript, forced miR-107 expression decreased luciferase activity by 33%, whereas, miR-107 mimics had no effect on the luciferase activity of the reporters containing a mutant lncRNA004787 3𲀲-UTR (Figure 4B). Therefore, we confirmed that lncRNA004787 was involved in weakening the expression of miR-107 and could further affect related biological function.

### LncRNA004787 was associated with the replication and proliferation of CVB3

**Figure 5:**
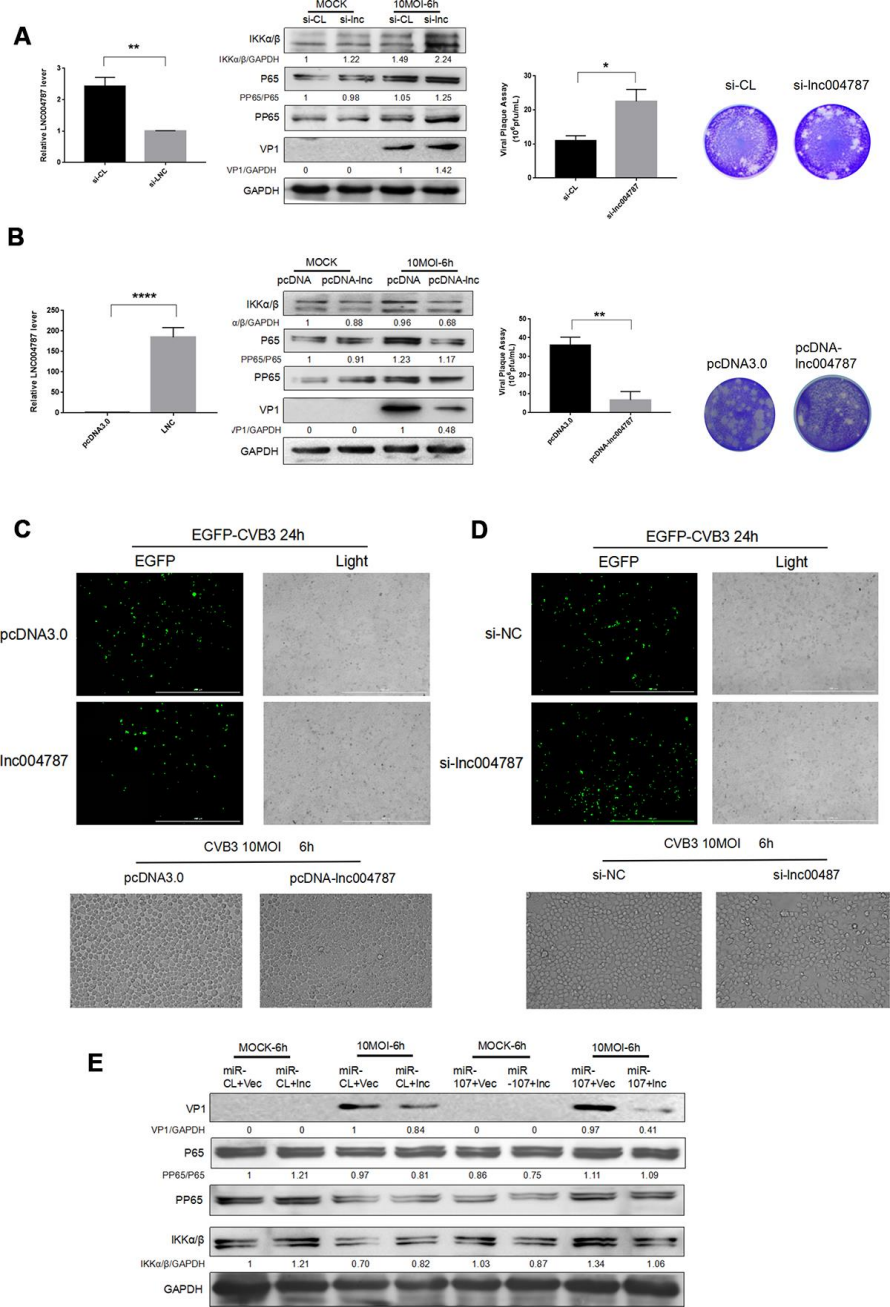
(**A**–**B**) After the knockdown or overexpression of IncRNA004787, RT-qPCR was performed for detecting its level changes. VP-1 and plaque assay were adopted for assessing CVB3 replication and virus release in Hela cells. (**C**–**D**) The replication level of EGFP-Iabeled CVB3 was observed in Hela cells which had been transfected with pcDNA-lncRNA004787 or si-lncRNA004787 (magnification, 4×). The morphology of the cells and the extent of the lesions were observed under a microscope (magnification, 100×). (**E**) The overexpression of IncRNA004787 partially reversed the facilitating effect of miR-107 on the production of VP-1 and the phosphorylation of NFκB. The activity of NFκB signaling was detected using western blot.

To further investigate the mechanism behind the effect of lncRNA004787 on CVB3, we transfected Hela cells with si-lncRNA004787, overexpressed vector pcDNA-lncRNA004787 in the cells, and performed CVB3 infection (10 MOI) for 48 h after the stable transfections. The RT-qPCR assay was performed to assess the knockdown and overexpression efficiency of lncRNA004787. Supernatant was collected and tested for the release capacity of CVB3 by viral plaque assay. The results showed that NF*κ*B phosphorylation was activated, CVB3 release was enhanced as viral replication significantly increased in si-lncRNA004787 group (Figure 5A). However, CVB3 replication was significantly inhibited, the viral release was weakened and NF*κ*B phosphorylation was suppressed when we overexpressed lncRNA004787 (Figure 5B). Therefore, it was believed that lncRNA0047887 had an incomplete inhibitory effect on CVB3 replication. We suspected that lncRNA004787 could inhibit the replication and release of CVB3 by other means.

In addition, EGFP-CVB3 was used to infect Hela cells which had been transfected with an empty vector or an overexpressed-lncRNA004787 vector. After the 24 h of infection, green fluorescence in lncRNA004787 group was significantly diminished compared with the control group under a fluorescence microscope, and so was CPE in the lncRNA004787 group (Figure 5C). Subsequently, Hela cells with lncRNA004787 knockdown and EGFP-CVB3 infection were observed. Compared with the control group, the green fluorescence intensity was significantly enhanced in the si-lncRNA004787 group, accompanied by the significant increase in CPE (Figure 5D).

For fully understanding the interaction between lncRNA004787 and miR-107 and their integrated effect on CVB3 replication, Hela cells were co-transfected with lncRNA004787 and miR-107 for 48 h. The expression of lncRNA004787 was determined by RT-qPCR analysis, and the expression of VP1 by western blot. The results showed that the level of lncRNA004787 in the experimental group (lncRNA004887+miR-107) was significantly lower than that in the control group (lncRNA004787+miR-CL) and the expression of VP1 in the experimental group was significantly higher than that in the control group (Figure 5E). This accentuated the antiviral effect of lncRNA004787, which was opposite to the biological function of miR-107. The experimental results further demonstrated the interaction between miR-107 and lncRNA004787. We concluded that lncRNA004787 was a upstream miRNA of miR-107 and was logically involved in inhibiting CVB3 replication and release, as well as the resulting inflammatory responses.

## DISCUSSION

The interaction between the virus and the host interferes viral replication and determines the pathogenesis of corresponding infectious diseases, which is regulated by a complex signaling network. MiRNA, as a regulator of RNA expressions, play an essential role in this interaction [20]. Previous studies have pointed out that miR-29a promotes hepatitis B virus (HBV) replication and expression by targeting SMARCE1 in hepatocellular carcinoma [21], and miR-204 and miR-126 inhibit HBV replication via two distinct mechanisms [22]. The above findings highlight the following two priorities: (i) the interaction between the virus and the host is diverse; (ii) the immune mechanism within the host cell can both inhibit and possibly facilitate viral replication. To explore the mechanism of how miR-107 regulates CVB3 replication, we have transfected the corresponding mimics and inhibitors, and find that it facilitates the replication and release of CVB3. The further experiments have confirmed two downstream target genes of miR-107, KLF4 and BACE1, and their binding site using the dual luciferase assay.

KLF4 encodes a protein belonging to the Kruppel transcription factor family. The encoded zinc finger protein is a requisite for the normal development of skin barrier function. Studies have figured out that KLF4 has an oncogene effect and can inhibit tumorigenesis in squamous cell carcinoma (SCC) cells by activating SMAD signaling pathway and SOX2 expression [23]. In our study, the level of KLF4 decreased after CVB3 infection in Hela cells, which means the function of cell membrane barrier has been impaired, creating favorable conditions for CVB3 replication and release.

BACE1 is involved in the formation of amyloid peptides. Despite its involvement in developing Alzheimer’s disease [24], it also associates with viruses. The protein encoded by BACE1 participates in the synthesis of amyloid precursor protein which interacts with HIV-1 [25].

However, roles of KLF4 and BACE1 in CVB3 replication remain unclear, so we have overexpressed them and discovered their negative regulations of CVB3 replication and proliferation. However, we hope to find the upstream lncRNA of miR-107 to get access to the precise mechanism. As a significantly novel RNA regulator, lncRNA is prominent in regulating the host antiviral immune response.

In 2011, WHO proposed a ceRNA theory [26]. Some scholars believe that the theory is quite suitable for ncRNAs, especially lncRNAs and circRNAs can bind to miRNAs more effectively and further regulate expressions of endogenous genes. In the existing studies, lncRNA H19, MALAT1 and MIR205HG as ceRNAs participate in the development of tumors [27–29]. In recent years, studies have also reported the association between lncRNA and viruses [30]. Hepatocytes infected with hepatitis C virus (HCV) can induce lncRNA-EGOT, and the latter increases viral replication by antagonizing antiviral responses [17]. HIV-1 infection can up-regulate the expression of lncRNA NEAT1 [18]. However, the induction of lncRNA after CVB3 infection still remains a puzzle.

The study has found a new lncRNA, namely lncRNA004787, located on chromosome 5. To further verify its regulation in the immune response of CVB3 infected hosts, we have overexpressed lncRNA004787 plasmid pcDNA-lncRNA004787 and find that lncRNA significantly inhibits CVB3 replication and slows down the cellular inflammatory responses, suggesting that it has antiviral properties. The co-transfection of lncRNA004787 and miR-107 has revealed that the antiviral effect of lncRNA004787 has been significantly repressed. Our study shows, for the first time, that lncRNA004787 effectively but incompletely inhibits the replication of CVB3. Based on the above experiments, it is reasonable to assume that miR-107 can increase CVB3 replication through the regulatory molecules KLF4, BACE1 and lncRNA004787. Therefore, we speculate that it may also exert its inhibitory effect indirectly through other endogenous genes.

However, a lncRNA may have multiple binding sites resulting in its binding to various miRNAs, and each miRNA can bind mRNAs of multiple genes to exert a negative regulatory effect. In this complex network of interactions, we have only studied one of them, and the other regulatory modes are still shrouded in mystery. The hypothesis of ceRNA will inspire more findings of mechanisms behind diseases. Though lncRNA is critical in the formation of ceRNA, whether the effect of lncRNA004787 on viral replication can be viewed from the perspective of ceRNA is a controversial point now and needs further experimental studies.

## MATERIALS AND METHODS

### Cell culture and viral infection

Hela cells were cultured with DMEM (Gibco, Grand Island, NY, USA) containing 10% fetal bovine serum (FBS). Cultured cells were washed with phosphate buffer saline (PBS) for 3 times, and starved Hela cells were incubated with serum-free medium for 1 h before CVB3 virus infection and supplemented with fresh medium for 1 h after infection. In addition, they were incubated at 37°C in a 5% CO_2_ incubator.

EGFP-CVB3 was provided by Professor Zhong Zhaohua from Harbin Medical University.

CVB3-Nancy was donated by Professor Chen Ruizhen from Shanghai Huashan Hospital.

### RNA extraction and RT-qPCR

Based on the manufacturer’s instructions, total RNA was extracted with RNA isolation reagent (Vazyme, Nanjing, China). RNA was transcribed using HiScript II, a one-step RT-qPCR kit. AceQ qPCR SYBR Green Master Mix was performed to determine levels of miRNAs by a relative quantitative method. U6 was used as the endogenous control to detect the content of miR-107 in CVB3-infected cells. The primer sequences were shown in the supplementary information (Table 1).

**Table 1:** Primer sequences.

**Primers**	**Sequences (5’-3’)**
miR-107 (Forward)	CTCAACTGGTGTCGTGGAGTCG
	GCAATTCAGTTGAGTGATAGCC
miR-107 (Reverse)	ACACTCCAGCTGGGAGCAGCATTGTACAGGG
U6 (Forward)	CTCGCTTCGGCAGCACA
U6 (Reverse)	AACGCTTCACGAATTTGCGT
KLF4 (Forward)	CTTTCCTGCCAGACCAGATG
KLF4 (Reverse)	GGTTTCTCGCCTGTGTGAGT
BACE1 (Forward)	ACAGCAACAGGGTGGTGGAC
BACE1 (Reverse)	TTTGAGGGTGCAGCGAACTT
GAPDH (Forward)	AGGTGAAGGTCGGAGTCAAC
GAPDH (Reverse)	GGGTGGAATCATATTGGAACA

### Western blot

Cell proteins were lysed in radioimmunoprecipitation assay (RIPA) buffer (Beyotime Biotechnology, Shanghai, China) containing phenylmethanesulfonyl fuoride (PMSF) and phosphatase inhibitors.

Proteins were separated by SDS-PAGE and then transferred to polyvinylidene difluoride (PVDF) membranes for antibody detection. The level of GAPDH was used as the internal parameter. The signal intensity of target protein bands was quantitated by grayscale scanning using the Image J program. All the experiments were carried out at least three times.

### Transfection of RNA and DNA

MiRNA, siRNAs and miRNA inhibitors were purchased from Gemma (Suzhou, China). According to the manufacturer’s instructions, we transfected Hela cells using Lipo3000 reagent. Samples were transfected for 48 h for further infection or RNA isolation.

We purchased the plasmid overexpressed BACE1 (human cDNA clone) (pcDNA3.1-bace1) and the plasmid overexpressed KLF4 (pCDNA3.1-KLF4) from Shanghai Sangon biotechnology Co., Ltd. (Shanghai, China).

### Viral plaque assay

The samples were diluted several times and added to Hela cells in a 6-well plate (8×10^5^ cells/m^2^). After the 1 h culture, cells were rinsed twice using PBS, and 2% low-melting AGAR medium was used to cover the culture for 72 h. The cells were fixed for 30 min in 75% ethanol and 25% glacial acetic acid and stained with crystal violet for 30 min. The virus plaques were counted in detail. All the tests were repeated at least three times.

### Constructs and dual-luciferase assay

According to the manufacturer’s instructions, the wild-type (wt) or mutant (mut) lncRNA004787 binding site was synthesized, and the pmiR-GLO double luciferase miRNA target expression vector (Promega, USA) was inserted after annealing. The oligonucleotides for annealing were listed in the supplementary information. The double-luciferase-reporter-gene-detection system (Vazyme, Nanjing, China) was adopted for double luciferase detection. Plasmids BACE1-miR107-mut, BACE1-miR107-wt and no-load pmiR-GLO were purchased from Suzhou Gemma Biotechnology Co., Ltd. (Suzhou, China).

### Statistical analysis

All experiments were repeated at least three times. Student’s *t* test was performed for paired comparisons among samples. Error bars represented as mean ± SD. A *p* value of < 0.05 (labeled with “*”) in two-tailed tests was considered as statistically significant, and “**” was used for labeling differences with the *p* value of < 0.01.
